# SilencerDB: a comprehensive database of silencers

**DOI:** 10.1093/nar/gkaa839

**Published:** 2020-10-12

**Authors:** Wanwen Zeng, Shengquan Chen, Xuejian Cui, Xiaoyang Chen, Zijing Gao, Rui Jiang

**Affiliations:** Ministry of Education Key Laboratory of Bioinformatics, Research Department of Bioinformatics at the Beijing National Research Center for Information Science and Technology, Center for Synthetic and Systems Biology, Department of Automation, Tsinghua University, Beijing 100084, China; College of Software, Nankai University, Tianjin 300071, China; Ministry of Education Key Laboratory of Bioinformatics, Research Department of Bioinformatics at the Beijing National Research Center for Information Science and Technology, Center for Synthetic and Systems Biology, Department of Automation, Tsinghua University, Beijing 100084, China; Ministry of Education Key Laboratory of Bioinformatics, Research Department of Bioinformatics at the Beijing National Research Center for Information Science and Technology, Center for Synthetic and Systems Biology, Department of Automation, Tsinghua University, Beijing 100084, China; Ministry of Education Key Laboratory of Bioinformatics, Research Department of Bioinformatics at the Beijing National Research Center for Information Science and Technology, Center for Synthetic and Systems Biology, Department of Automation, Tsinghua University, Beijing 100084, China; Ministry of Education Key Laboratory of Bioinformatics, Research Department of Bioinformatics at the Beijing National Research Center for Information Science and Technology, Center for Synthetic and Systems Biology, Department of Automation, Tsinghua University, Beijing 100084, China; Ministry of Education Key Laboratory of Bioinformatics, Research Department of Bioinformatics at the Beijing National Research Center for Information Science and Technology, Center for Synthetic and Systems Biology, Department of Automation, Tsinghua University, Beijing 100084, China

## Abstract

Gene regulatory elements, including promoters, enhancers, silencers, etc., control transcriptional programs in a spatiotemporal manner. Though these elements are known to be able to induce either positive or negative transcriptional control, the community has been mostly studying enhancers which amplify transcription initiation, with less emphasis given to silencers which repress gene expression. To facilitate the study of silencers and the investigation of their potential roles in transcriptional control, we developed SilencerDB (http://health.tsinghua.edu.cn/silencerdb/), a comprehensive database of silencers by manually curating silencers from 2300 published articles. The current version, SilencerDB 1.0, contains (1) 33 060 validated silencers from experimental methods, and (ii) 5 045 547 predicted silencers from state-of-the-art machine learning methods. The functionality of SilencerDB includes (a) standardized categorization of silencers in a tree-structured class hierarchy based on species, organ, tissue and cell line and (b) comprehensive annotations of silencers with the nearest gene and potential regulatory genes. SilencerDB, to the best of our knowledge, is the first comprehensive database at this scale dedicated to silencers, with reliable annotations and user-friendly interactive database features. We believe this database has the potential to enable advanced understanding of silencers in regulatory mechanisms and to empower researchers to devise diverse applications of silencers in disease development.

## INTRODUCTION

One of the main challenges for research in genomics is to identify functional elements in the genome (1), especially gene regulatory elements that play a vital role in transcriptional regulation, cell differentiation, tissue homeostasis and disease development (2,3). Gene regulatory elements, including promoters, enhancers, silencers, etc, are short regions of non-coding DNA sequences that reside in open chromatin in a cell type-specific manner and are bound by sets of transcription factors (TFs) for positive or negative transcriptional control (4,5). Among various classes of regulatory elements, the research community has thus far been favoring enhancers over the past few decades (6) for their ability to activate gene expression, encouraging the emergence of various enhancer resources, including validated enhancer databases (7,8), comprehensive enhancer databases (9,10), super-enhancer databases (11,12), validated enhancer-disease databases (13,14), enhancer prediction methods (15,16) and enhancer-promoter interaction prediction methods (17,18). On the other hand, the roles of silencers in the downregulation of gene expression were first identified around 30 years ago but much less has been studied about these *cis*-regulatory elements than their enhancer counterparts (19,20).

Silencers are known to regulate distally located genes by forming silencer-promoter interactions and suppress mRNA expression from target promoters (21,22). The negative regulatory mechanism of silencers, together with the positive one of enhancers, can constitute a more holistic perspective to understand gene transcriptional control (23–25). However, few transcriptional silencers have been identified and they remain poorly understood. Only recently, several studies successfully characterized silencers in human, mouse and drosophila by efficient functional assays on a genomic scale and provided novel understanding of their regulatory mechanisms, bringing silencers into the spotlight (26–30). The observation that genetic variants identified from genome-wide association studies (GWASs) are enriched in cell type-specific silencer regions further emphasizes the importance of silencers (27,28). Some silencers are also demonstrated to be bifunctional and capable of enhancing gene expression in other cellular contexts, challenging the common practice of treating enhancer and silencer as separate classes (26,31). All these findings have made the identification and characterization of silencers an important premise for future research.

Hence, to facilitate future studies of silencers, we developed SilencerDB (http://health.tsinghua.edu.cn/silencerdb/ or http://bioinfo.au.tsinghua.edu.cn/silencerdb/), a comprehensive database of silencers. The current version of SilencerDB contains 33 060 validated silencers and 5 045 547 predicted silencers for 268 cell lines across eight species. Among the validated silencers, 32 707 were manually curated from high-throughput experiments (e.g. MPRA, CRISPR) and 353 from low-throughput experiments (e.g. transient transfection assays, reporter assays). Predicted silencers are identified via the correlation-based model (29), the SVM-based model (13), the gkmSVM-based model (30) and our newly developed deep learning-based model DeepSilencer (https://github.com/xy-chen16/DeepSilencer). Each silencer entry in SilencerDB is annotated with extensive details including general silencer information and potential regulatory gene information (e.g. the nearest gene and PECA (32) predicted regulatory genes). Other functionalities in SilencerDB include intuitive and hierarchical browsing, advanced searching, interactive visualization with custom tracks, data downloading in different formats and detailed statistical analysis. We expect that SilencerDB will provide valuable resources of silencers, enable deeper insights into gene regulatory mechanisms and aid in developing downstream applications.

## DATA COLLECTION AND DATABASE CONTENT

### Data collection

**Figure 1.**
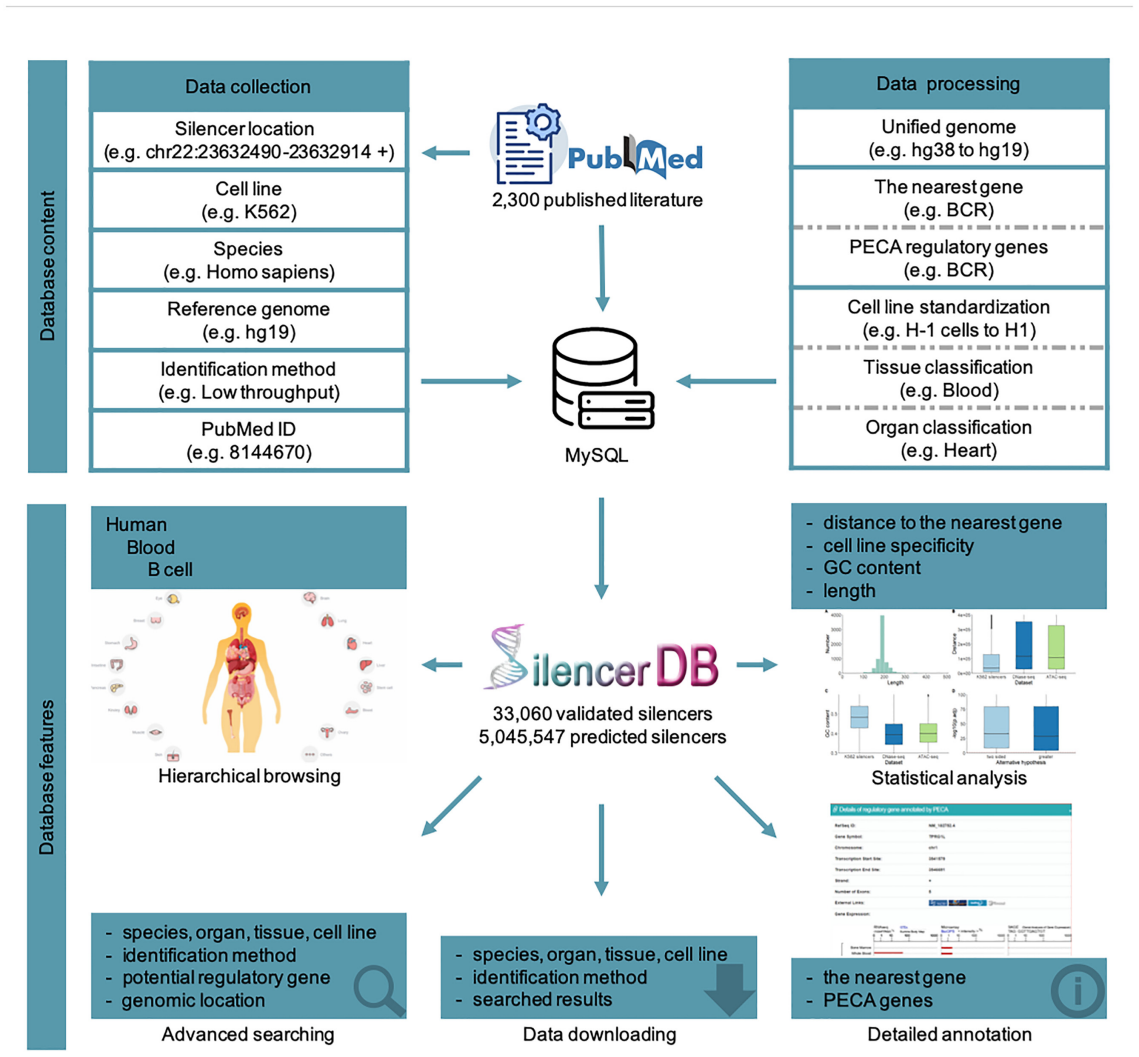
Overview of data collection, data processing and annotation, and database features of SilencerDB.

For the collection of silencers, we adopted a series of standardized procedures to ensure consistent and reliable data collection (33–35). First, a total of 2300 abstracts with the keyword ‘silencer’ were retrieved from the PubMed database by June 2020. These candidate articles were then filtered based on availability of genomic locations of silencers and the form of identification. Validated silencers were identified by either high-throughput or low-throughput experimental techniques such as MPRA, CRISPR, transient transfection assays and reporter assays. Predicted silencers were collected using the correlation-based model (29), the SVM-based model (17), the gkmSVM-based model (30), and our newly developed deep learning-based model DeepSilencer. The current release of the database contains silencers retrieved from a total of 456 articles related to validated silencers and three articles related to predicted silencers. The full text of each candidate article was manually reviewed in detail by at least two independent researchers to extract the information of silencers. Each entry contains general information such as species, cell line, reference genome, genomic location, PubMed ID of the publication as well as details about the experimental or computational method used for its identification (Figure 1).

### Data processing and annotation

All collected silencers were mapped to a specific reference genome (Table 1) by liftOver (36) (e.g. GRCh37/hg19 for *Homo sapiens* and GRCm37/mm9 for *Mus musculus*) to ensure the unification of genomic locations of silencers. 2048 predicted silencers and five validated silencers whose genomic locations cannot be converted into the specific reference genome were omitted (Figure 1).

**Table 1.** Versions of reference genomes

Species	Genome
*Homo sapiens*	GRCh37/hg19
*Mus musculus*	GRCm37/mm9
*Rattus norvegicus*	Rnor_6.0
*Drosophila melanogaster*	Dmel_Release_6
*Bubalus bubalis*	ASM312139v1
*Sus scrofa*	Sscrofa11
*Canis lupus familiaris*	CanFam3.1
*Gallus gallus*	galGal6

Each silencer in SilencerDB was annotated with extensive details for categorization. A list of distinct cell lines was first extracted from original reviewed articles. After standardizing the names of cell lines into the standard list from ENCODE (37) and removing those with ill-formed names, we further classified standardized cell lines into respective tissues and organs. Silencers were grouped into a cell line, tissue, organ and species hierarchical structure according to their localization, morphology and functionality. Entries with no specified source of cell line, tissue or organ were recorded as ‘Others’ (Figure 1).

To further facilitate the study of gene regulatory mechanisms, each silencer was annotated with the nearest gene as well as potential regulatory genes. The nearest gene of a specific silencer was annotated according to its genomic location. The potential genes which negatively interact with a specific silencer were retrieved from the original articles or predicted by the PECA model which infers gene regulatory relations using matched expression and accessibility data across diverse cellular contexts (32).

### Database statistics

**Figure 2.**
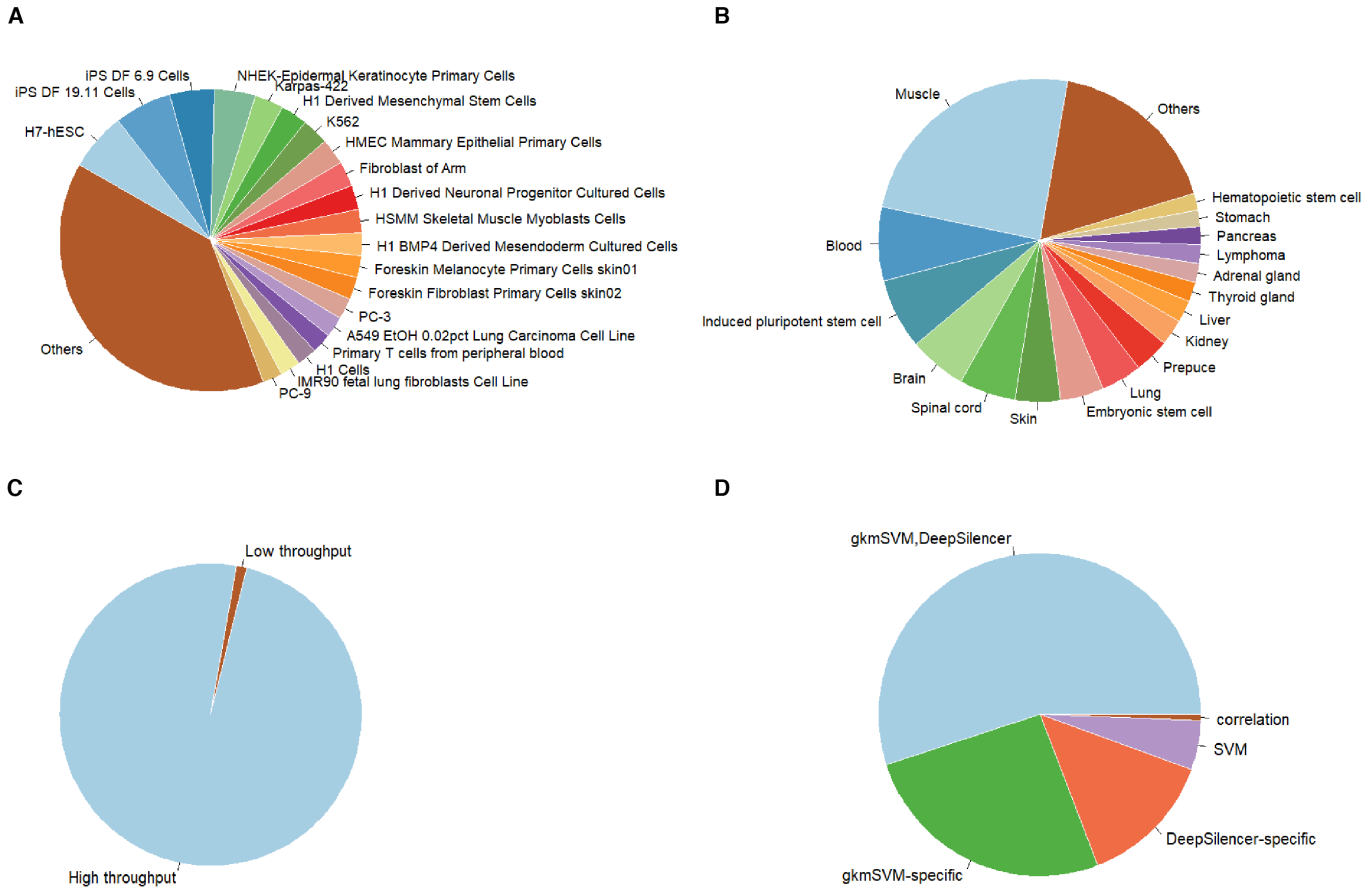
Statistics of silencers in SilencerDB. (**A**) Number distribution of human silencers across different cell lines. (**B**) Number distribution of human silencers across different tissues. (**C**) Number distribution of silencers across different experimental methods. (**D**) Number distribution of silencers across different predicted models.

The current version of SilencerDB contains 33 060 validated silencers and 5 045 547 predicted silencers. The detailed statistics of SilencerDB, including the number of silencers, the number of organs, tissues and cell lines in different species, are shown in Table 2. In particular, SilencerDB contains 3 561 242 and 1 517 324 silencers in human and mouse, respectively. In human, validated silencers are derived from 15 organs, 27 tissues and 126 cell lines, and predicted silencers are derived from 16 organs, 38 tissues and 83 cell lines. In mouse, validated silencers are derived from 3 organs, 12 tissues and 28 cell lines, and predicted silencers are derived from 9 organs, 10 tissues and 17 cell lines. As shown in Figure 2A, B, most of human silencers are derived from muscle tissue (24.4%) and the H7-hESC cell line (6.2%). Most of mouse silencers are derived from brain tissue (58.1%) and the MEL cell line (8.0%) (Supplementary Figure S1A, B).

**Table 2.** Statistical information of SilencerDB

	Method	*Homo sapiens*	*Mus musculus*	Others	Total
Silencers	Validated	8827	24 192	41	33 060
	Predicted	3 552 415	1 493 132	0	5 045 547
Organs	Validated	15	3	10	28
	Predicted	16	9	0	25
Tissues	Validated	27	12	10	49
	Predicted	38	10	0	48
Cell lines	Validated	126	28	23	177
	Predicted	83	17	0	100

The silencers were collected from several different experimental methods and predicted models. Validated silencers were collected from high-throughput methods (98.93%) and low-throughput methods (1.07%) (Figure 2C). Predicted silencers were collected using several state-of-the-art models, including the correlation-based model (0.59%), the SVM-based model (4.92%), the gkmSVM-based model (80.87%), and our DeepSilencer model (68.66%) (Figure 2D). Notably, 55.04% of the silencers were collected from both the gkmSVM-based model and the DeepSilencer model. A brief introduction of DeepSilencer and a comparison between silencers predicted by the gkmSVM-based model and those predicted by the DeepSilencer model can be found in Supplementary Notes and Supplementary Figure S2.

## DATABASE FEATURES AND APPLICATIONS

### User-friendly browsing

**Figure 3.**
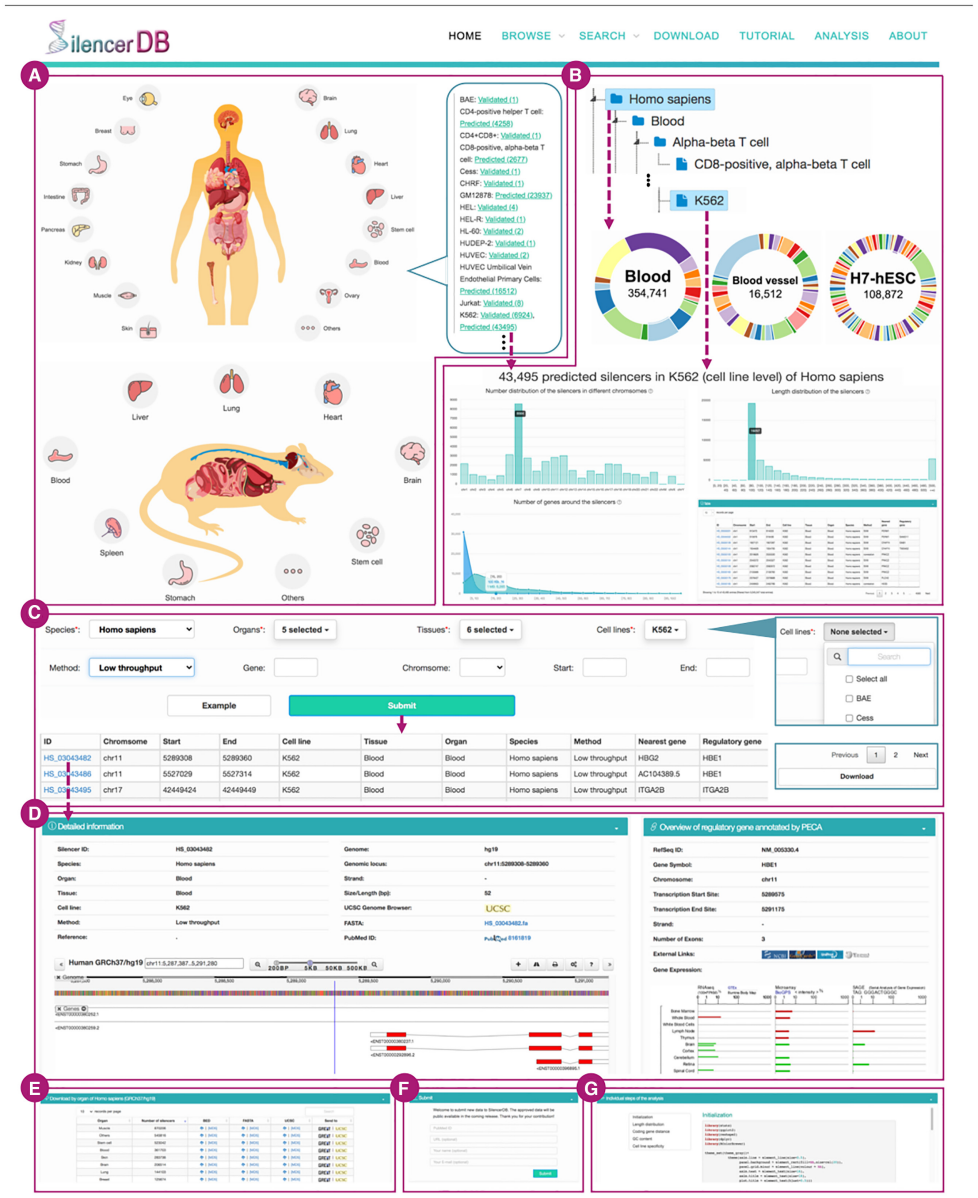
The schematic features on various webpages of SilencerDB. (**A**) *Home:* interactive images of human and mouse anatomy (**B**) *Browse*: hierarchical categorization of silencers and comprehensive statistics with respect to the selected subset of silencers. (**C**) *Search*: various options and filters for searching the silencers of interest. (**D**) *Detail:* detailed information of each silencer entry (**E**) *Download*: multiple standard-compliant download options with external links. (**F**) *About*: silencer submission portal. (**G**) *Analysis*: exemplary case study of silencers.

We built an intuitive web interface for researchers to explore and analyze silencers. Interactive images of human and mouse anatomy displayed on the *Home* page (Figure 3A) allow direct access to validated or predicted silencers relevant to cell lines in different organs of species. In detail, after clicking one of the organ icons, users can access relevant cell lines in a pop-up window and browse validated or predicted silencers of interest by clicking the corresponding hyperlinks. Users can also explore silencers of interest via the *Browse* page. To assist in the selection process, we provide a tree-based hierarchical structure in the left panel and a comprehensive set of statistics with respect to the selected subset. As shown in Figure 3B, the statistics include (i) the number distribution of silencers across various organs, tissues and cell lines, (ii) the number distribution of silencers in different chromosomes, (iii) the length distribution of silencers and (iv) the number distribution of silencers in discrete intervals of neighboring (100K or 1M base-pairs upstream and downstream of the silencer) gene counts. The basic information of the selected subset of silencers is displayed in an interactive table, where each row denotes a silencer, and columns consist of the silencer ID maintained by our database, genomic location, cell line, tissue, organ, species, the method used to identify the silencer, the nearest gene and the potential regulatory gene annotated by the PECA model. Users can click the silencer ID to access the detailed information on a new webpage.

### Advanced searching

On the *Search* page, the user-interface offers ample search options and filters in the drop-down menus (Figure 3C). Users can optionally specify the scope of a query by determining species, tissue, cell line, the identification method, the nearest or regulatory gene, and genomic location for the silencers of interest. A checked select-all option means ignoring the corresponding filter for the item. Besides, an exemplary search entry can be generated by clicking on the ‘Example’ button. The search results will be displayed on an interactive table similar to that on the *Browse* page and users can access the detailed information on a new webpage by clicking the silencer ID. An option to download the search results locally is given at the bottom right corner of the page.

### Detailed information

Selected entries from the *Browse* or *Search* page are compiled in a tabular format with each silencer ID linked to the *Detail* page of the entry. As shown in Figure 3D, the *Detail* page encompasses various attributes associated with the selected silencer including the silencer ID, category (by species, organ, tissue and cell line), identification method, reference and corresponding PubMed ID, reference genome, genomic location, length, external link to UCSC and FASTA file. An embedded interactive and smooth genomics visualization tool, Biodalliance (38) is incorporated, from which users can study the proximity information of the silencer and have the flexibility to add, configure, export genome tracks for quick analysis. We also designed overviews of the annotated nearest gene and the potential regulatory genes, including their gene symbols, chromosome, transcription start and end sites, gene expression in various tissues. More information about the genes are available through several links to external resources, such as NCBI (39), GeneCards (40), UniProt (41) and Wikipedia.

### Data download and submission

For downloading silencers, we refer users to the *Download* page where we group silencers by species, organ, tissue, cell line and identification method, and provide dataset in multiple standard-compliant formats, including BED, FASTA and UCSC custom track (Figure 3E). Users can verify the integrity of a downloaded file with the corresponding MD5 checksum file. Moreover, each batch of silencers is coupled with two links, one to the UCSC Genome Browser (36) with an automatically added custom track for visualization, and one to the Genomic Region Enrichment of Annotation Tool (GREAT) (42) for functional prediction of the silencers by analyzing significant pathways and generating statistically associated annotations.

Lastly, on the *About* page, users can share new silencer data by submitting the PubMed ID, the accessible URL of the data, and the contributor information (Figure 3F). Through an internal standardized collection and verification procedure, additional silencer data will be compiled and published in the next stable release within a maximum of 6 months.

### A case application on the analysis of silencers of interest

As SilencerDB is the first database on silencers, we present a supplementary case study on the *Analysis* page to demonstrate a preliminary analysis of silencers (Figure 3G). Taking silencers validated in the human K562 cell line as an example, the lengths of the silencers are mainly between 100 and 300 bp (Supplementary Notes, Supplementary Figure S3A). The average distance from a silencer to its nearest coding gene is 61 721 bp, which is less than that from a DNase-seq peak (144 767 bp) or an ATAC-seq peak (132 892 bp) (Supplementary Notes, Supplementary Figure S3B). On average, the GC content of a silencer is 0.4896, which is higher than that of a DNase-seq peak (0.4266) or an ATAC-seq peak (0.4222) (Supplementary Notes, Supplementary Figure S3C).

We next investigated cell line specificity of silencers by checking whether silencers validated in a cell line show a higher degree of chromatin accessibility (openness score) in the same cell line than in other cell lines. Taking validated silencers in human K562 cell line as an example, we first used OPENANNO (http://health.tsinghua.edu.cn/openness/anno/) to efficiently calculate openness scores of these silencers across 199 human cell lines. To check whether these silencers have higher openness scores in the K562 cell line than those in other 198 cell lines, we performed a one-sided Wilcoxon test for silencer openness scores in K562 and the remaining 198 cell lines and obtained 198 FDR *P*-values (Benjamini and Hochberg correction) respectively. Results, as shown in Supplementary Figure S3D, demonstrate that these K562 silencers indeed have higher openness scores in the K562 cell line than those in the other 162 cell lines (FDR = 0.05), suggesting the cell line specificity of silencers (27,30).

## SYSTEM DESIGN AND IMPLEMENTATION

The SilencerDB website is maintained on a Linux-based Apache web server (https://www.apache.org). The web-frontend interface uses Bootstrap v3.3.7 framework (https://getbootstrap.com/docs/3.3/) for display optimization. Plug-ins for the jQuery and JavaScript library, including DataTables v1.10.19 (https://datatables.net), Biodalliance v0.13.8 (http://www.biodalliance.org/about.html), and morris.js v0.5.0 (https://morrisjs.github.io/morris.js/index.html), are used to implement advanced tables, genome browser, and charts, respectively. The server-backend uses PHP v7.4.5 (http://www.php.net). All data is stored in a MySQL v8.0.20 (http://www.mysql.com) database. The current version supports most of mainstream web browsers, such as Google Chrome, Firefox, Opera, Microsoft Edge, Apple Safari, etc.

## CONCLUSION

Although the study of transcriptional regulation is one of the most research-intensive topics in biology, only now we start to interpret the underlying logic of regulatory elements systematically, thanks to advancement in both experimental and computational techniques. Though regulatory elements are known to be able to induce either positive or negative transcriptional control (43–45), the community for the past few decades has been mostly studying enhancers which amplify transcription initiation upon binding of transcription factors (46), with less emphasis given to silencers which prevent the transcription initiation and repress gene expression (47). Recent breakthroughs in silencer research brings these negative cis-regulatory elements into spotlight (48–50). While there have been numerous databases and downstream applications for enhancers, the lack of resources for silencer identification could be a problematic hindrance for future research.

Therefore, to fulfil the gap, we developed SilencerDB which, to the best of our knowledge, is the first comprehensive database at this scale dedicated to silencers. With systematic data collection procedures, standardized datasets as well as a user-friendly web platform designed for research standards, we hope that SilencerDB can benefit biologists and data scientists to achieve better understanding of the role of silencers in regulatory mechanisms and empower them to construct more comprehensive gene regulatory networks by combining negative regulatory mechanisms of silencers with the positive ones of enhancers. Moreover, since GWAS-identified risk variants in non-coding regions of the genome exert phenotypic effects through perturbation of transcriptional gene promoters, enhancers and silencers etc., SilencerDB have the potential to give insights to a more complete interpretation of GWAS risk variants and aid in developing new approaches for disease prevention and treatment (51–53).

After the first release of SilencerDB, one of our plans for the immediate future is to incorporate more comprehensive epigenomic annotations, such as different types of transcription factor binding, histone modification and chromatin accessibility annotation in different cell lines. In order to expedite the collection process and expand our dataset, we would also like to incorporate a web-based tracking and data entry system to carry out a bi-weekly PubMed search to identify silencer-related research. Other areas that the team hope to improve on include better visualization tools for silencer comparisons and speed-up for search queries.

## DATA AVAILABILITY

Users can access any feature available in the database without the need to register or login. All data is freely accessible to the research community at http://health.tsinghua.edu.cn/silencerdb/ or http://bioinfo.au.tsinghua.edu.cn/silencerdb/. In addition to various options for downloading data on the *Download* page, users can customize and download the filtered data on the *Search* page.

## Supplementary Material

gkaa839_Supplemental_File
