# Supplementary material for: SilencerDB: a comprehensive database of silencers
Source: Nucleic Acids Res. 2020 Oct 12;49(D1):D221–8. doi: 10.1093/nar/gkaa839 (PMC7778955; doi:10.1093/nar/gkaa839)
Supplement: gkaa839_Supplemental_File [file gkaa839_supplemental_file.pdf]

Silencer DB: a comprehensive database of silencers

Wanwen Zeng[2,†], Shengquan Chen[1,†], Xuejian Cui[1,†], Xiaoyang Chen[1], Zijing Gao[1] and Rui Jiang[1,*]

[1] Ministry of Education Key Laboratory of Bioinformatics, Research Department of Bioinformatics at the Beijing National Research Center for Information Science and Technology, Center for Synthetic and Systems Biology, Department of Automation, Tsinghua University, Beijing 100084, China
[2] College of Software, Nankai University, Tianjin 300071, China

* To whom correspondence should be addressed. Tel: +86 10 6279 5578; Email: ruijiang@tsinghua.edu.cn

† The authors wish it to be known that, in their opinion, the first 3 authors should be regarded as joint First Authors

Supplementary Notes

Brief introduction of DeepSilencer

For accurate classification of silencers, we proposed a deep learning-based model named DeepSilencer, which consists of four modules. First, a pre-processing module transforms DNA sequences into one-hot encoded matrices and vectors of *k*-mers counts. Second, a convolutional neural network (CNN) module uses multiple convolutional and pooling layers to extract features from the one-hot encoded DNA sequences. Third, an artificial neural network (ANN) module is adopted to sufficiently learn features from *k*-mers. Finally, a joint module integrates outputs of the CNN and ANN modules to predict whether the input sequence is a silencer or not. The detailed network structure and hyper-parameters of DeepSliencer are shown in https://github.com/xy-chen16/DeepSilencer. Following Jayavelu *et al*. (1), we collected the uncharacterized cis-regulatory elements (CREs) in K562 cell line from MPRA provided by the authors. We chose the top 2,000 uncharacterized CREs sequences with the lowest MPRA activity as the positive set, and the bottom 2,000 uncharacterized CREs with highest MPRA activity as the negative set. We randomly selected 80% of the data as the training set and used the remaining 20% of data as the testing set. DeepSilencer accurately predicted the uncharacterized CREs on the test set, and achieved superior classification performance than the gapped *k*-mer SVM (gkmSVM) (1). The area under the receiver operating characteristic (AUROC) was 0.827 for DeepSilencer while it was 0.81 for gkmSVM and the area under the precision-recall curve (AUPRC) was 0.842 for DeepSilencer while 0.76 for gkmSVM.

Detailed comparison between DeepSilencer and gkmSVM

We further performed comparative analyses between silencers predicted in human K562 cell line by the gkmSVM-based model and those predicted by the DeepSilencer model. The length distributions of silencers

predicted by the gkmSVM-based model and our DeepSilencer model are shown in Figure S2A. The length distribution of silencers predicted by DeepSilencer is slightly different from that predicted by gkmSVM (two-sided Wilcox test *p*-value=0.0007). Other results showed no significant difference between silencers predicted by DeepSilencer and gkmSVM in terms of the distance between silencers and the nearest coding genes (two-sided Wilcox test *p*-value=0.5209, Figure S2B), the GC content of silencers (two-sided Wilcox test *p*-value=0.9299, Figure S2C), and the chromatin accessibility of silencers (two-sided Wilcox test *p*-value=0.5699, Figure S2D).

Since we used the same training set as gkmSVM, most of the silencers predicted by gkmSVM (68.07%) overlap with that by our DeepSilencer model. To examine the functionality of unique silencers predicted by DeepSilencer model, we took HS_03109236 (chr3: 48,997,302-48,997,398) as example. HS_03109236 overlaps with a K562 validated silencer, HS_00278862 (chr3: 48,997,266-48,997,466). Some epigenomic tracks of HS_00278862 and HS_03109236 are presented in Figure S2E, showing that the region of this silencer was open and marked by some repressive histone modifications. This example indicates the potential validation hints of this predicted silencer.

Comparison of validated silencers and other open regions in the human K562 cell line

To investigate the difference between validated silencers and other open regions, we systematically compared validated silencers and other open regions in human K562 cell line. We obtained two datasets of open regions in K562 (DNase-seq peaks and ATAC-seq peaks) from NCBI as baseline. The length distributions of validated silencers and that of open regions in K562 are shown in Figure S3A. The lengths of silencers are mainly between 100~300bp, while the lengths of open regions are mainly between 200~400 bp for the DNase-seq dataset and 600~1000 bp for the ATAC-seq dataset (Figure S3A). The distributions of distance from silencers and open regions to corresponding nearest genes are shown in Figure S3B. The average distance from a silencer to its nearest coding gene is 61,721 bp, which is significantly less than that from a DNase-seq peak to its nearest coding gene (144,767 bp, one-sided Wilcox test *p*-value<2.2e-16)) and from an ATAC-seq peak to its nearest coding gene (132,892 bp, one-sided Wilcox test *p*-value<2.2e-16)). The GC content distribution of regions in different datasets is shown in Figure S3C. On average, the GC content of validated K562 silencers is 0.4896, which is much higher than that of DNase-seq peaks (0.4266, one-sided Wilcox test *p*-value<2.2e-16) and ATAC-seq peaks (0.4222, one-sided Wilcox test *p*-value<2.2e-16). We performed one-sided Wilcoxon test and two-sided Wilcoxon test for silencer openness scores in K562 against the remaining 198 cell lines and obtained 198 FDR *p*-values using Benjamini and Hochberg correction respectively. The one-sided and two-sided p-value distributions are shown in Figure S3D, suggesting the cell line specificity of silencers.

References

1. Doni Jayavelu, N., Jajodia, A., Mishra, A. and Hawkins, R.D. (2020) Candidate silencer elements for the human and mouse genomes. *Nat Commun*, **11**, 1061.

Supplementary Figures

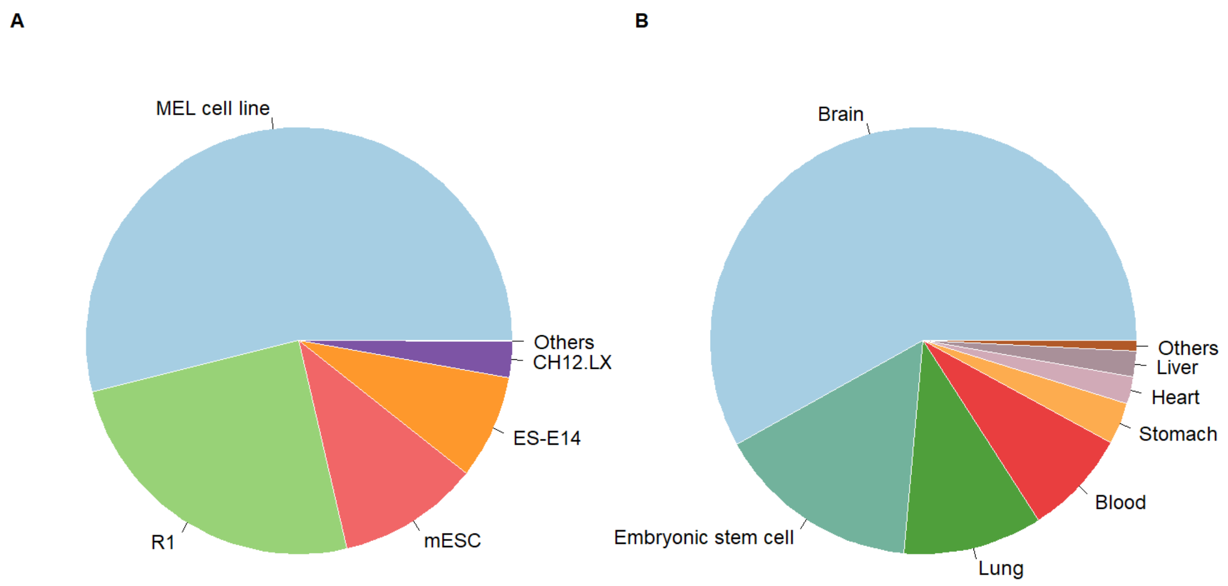

Supplementary Figure S1. Number distribution of silencers across different mouse cell lines/tissues. (A) Number distribution of silencers across different mouse cell lines. (B) Number distribution of silencers across different mouse tissues.

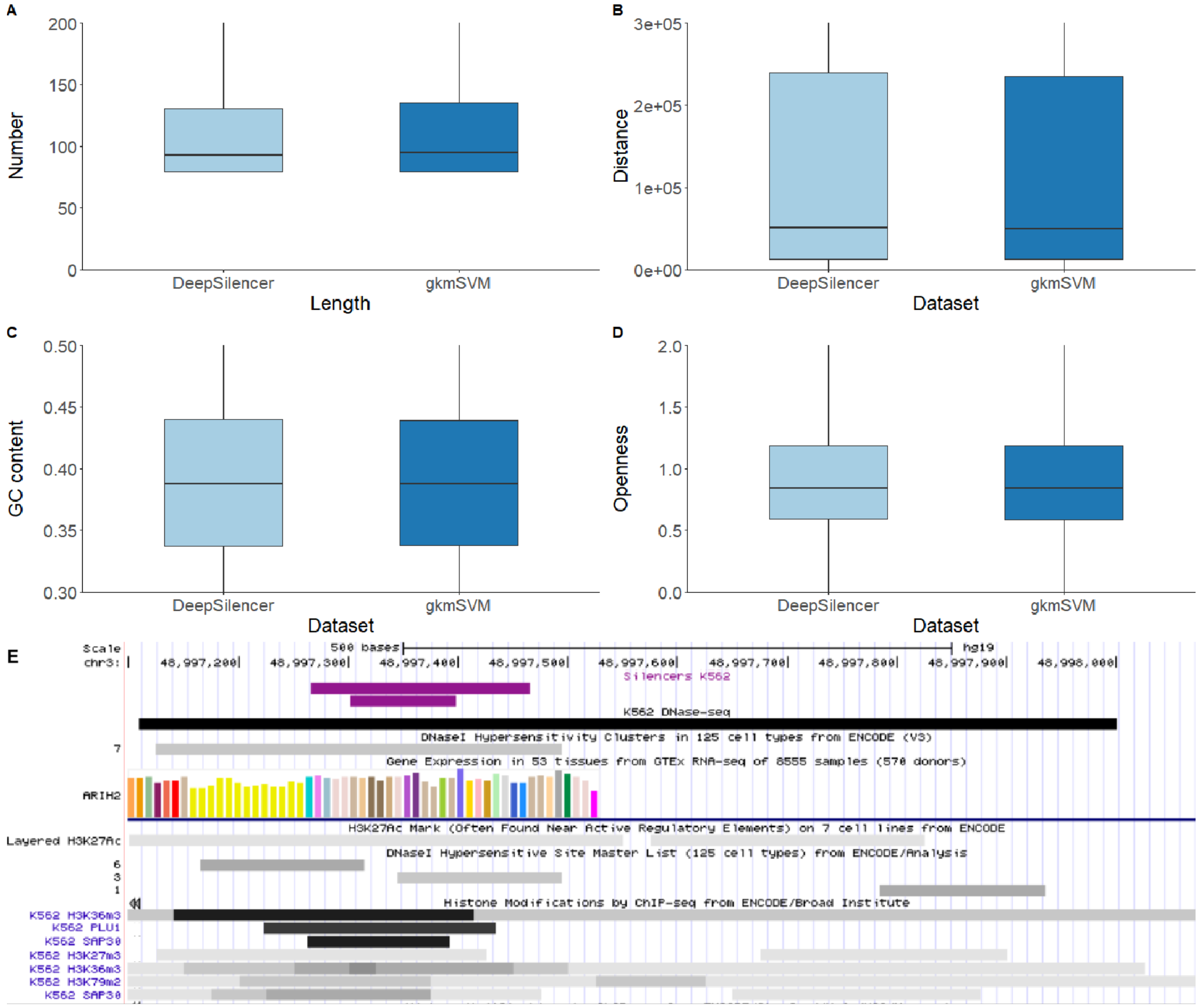

Supplementary Figure S2. Comparison of silencers predicted by gkmSVM-based and DeepSilencer. (A) The length distribution of silencers. (B) The distribution of distance between silencers and corresponding nearest genes. (C) The GC content distribution of silencers. (D) The chromatin accessibility (openness scores from OPENANNO) distribution of silencers. (E) Visualization of the surrounding regions of HS_00278862 (chr3: 48,997,266-48,997,466) and HS_03109236 (chr3: 48,997,302-48,997,398) in UCSC browser.

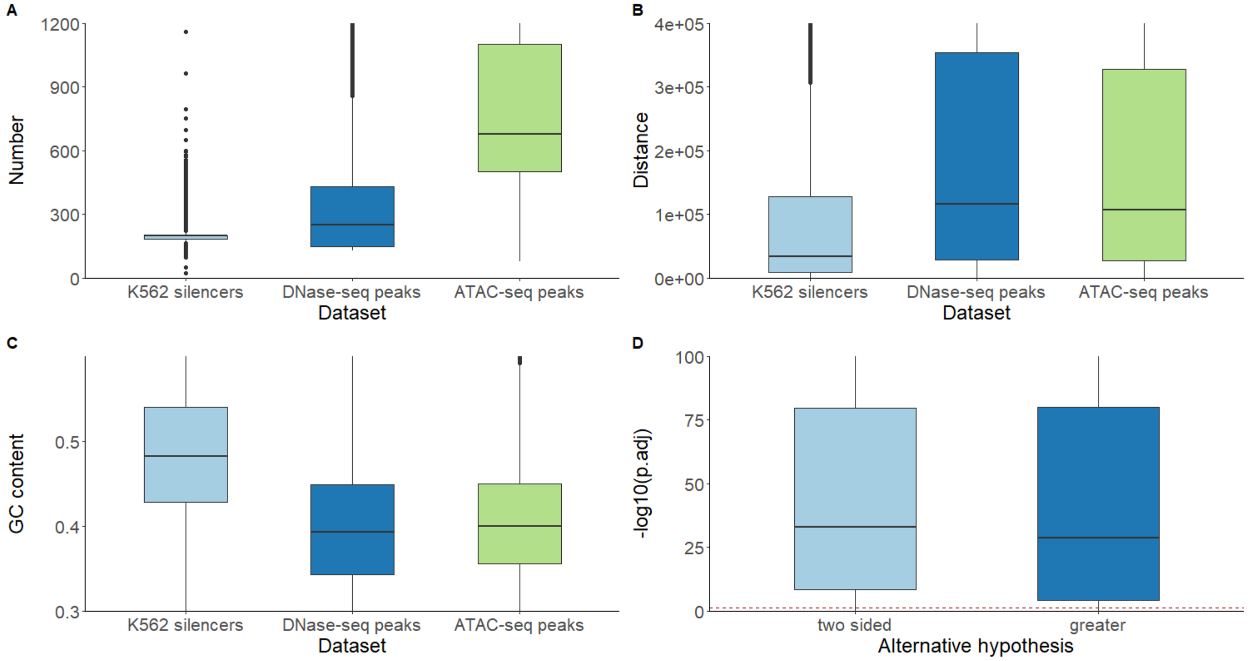

Supplementary Figure S3. Comparison of validated silencers and open regions in the human K562 cell line. (A) The length distribution of silencers and open regions. (B) The distribution of distance to the corresponding nearest genes. (C) The GC content distribution of silencers and open regions. (D) Comparison of the chromatin accessibility between silencers and open regions.
